# Neurotrophic Keratitis Due to Congenital Corneal Anesthesia with Deafness, Hypotonia, Intellectual Disability, Face Abnormality and Metabolic Disorder: A New Syndrome?

**DOI:** 10.3390/medicina58050657

**Published:** 2022-05-13

**Authors:** Arvydas Gelzinis, Dovile Simonaviciute, Agne Krucaite, Luca Buzzonetti, Hélène Dollfus, Reda Zemaitiene

**Affiliations:** 1 Department of Ophthalmology, Medical Academy, Lithuanian University of Health Sciences, 44037 Kaunas, Lithuania; arvydas.gelzinis@kaunoklinikos.lt (A.G.); agne.krucaite@kaunoklinikos.lt (A.K.); reda.zemaitiene@kaunoklinikos.lt (R.Z.); 2 Ophthalmology Department, Bambino Gesù Children’s Hospital, IRCCS, 00165 Rome, Italy; luca.buzzonetti@opbg.net; 3 Centre de Référence Pour les Affections Rares en Génétique Ophtalmologiques, Hôpitaux Universitaires de Strasbourg, F-67000 Strasbourg, France; helene.dollfus@chru-strasbourg.fr; 4 Laboratoire de Génétique Médicale, INSERM U1112, Institut de Génétique Médicale d’Alsace (IGMA), Université de Strasbourg, F-67000 Strasbourg, France

**Keywords:** neurotrophic keratitis, neurotrophic keratopathy, congenital corneal anesthesia, corneal erosion, corneal ulcer, trigeminal nerve

## Abstract

Neurotrophic keratitis is a rare degenerative disease of the cornea that can lead to corneal ulceration, scarring, and significant visual impairment. It most commonly occurs in adults and is rarely diagnosed in children. Congenital corneal anesthesia is an extremely rare condition that requires appropriate ophthalmologists’ attention in making diagnosis and treatment decisions. This condition usually presents in infancy or early childhood and is characterized by rare blinking rate, decreased tearing or a corneal ulcer that is unresponsive to treatment. In this case report, we describe a patient with multiple systemic and neurological disorders who presented to the ophthalmology department due to corneal erosion unresponsive to treatment. Brain magnetic resonance imaging confirmed bilateral trigeminal hypoplasia and the diagnosis of neurotrophic keratopathy due to bilateral congenital corneal anesthesia was made. The discrepancy between clinical signs and symptoms or treatment non-response in cases of corneal erosions should alert the ophthalmologists to suspect trigeminal dysfunction. MRI is the gold standard to confirm congenital corneal anesthesia and to differentiate from other possible neurotrophic keratitis causes.

## 1. Introduction

The cornea is the most densely innervated tissue in the human body. Innervation is supplied by the ophthalmic branch of the trigeminal nerve. Corneal nerves are responsible for reflex tearing, blinking and the release of trophic factors, such as nerve growth factor, substance P, calcitonin, gene-related peptide, neuropeptide Y, and acetylcholine [1]. Blinking and tearing reflexes are important to protect corneal surface from environmental factors and to provide the essential nutrients and oxygen to the cornea. All of these factors jointly promote epithelial cell proliferation, migration, adhesion, differentiation and are necessary for the structural and functional integrity of the ocular surface [1]. Damage to the corneal nerves causes the loss of corneal sensation and trophic functions which consequently results in neurotrophic keratitis [2]. The function of the trigeminal nerve can be disturbed by systemic or ocular, congenital or acquired diseases or iatrogenic lesions. Despite the underlying cause, neurotrophic keratitis presents as a punctate keratitis that may progress to persistent epithelial defect or corneal ulcer. The diagnosis is mainly based on clinical history, decreased corneal sensitivity, and the presence of corneal damage [2,3]. Congenital corneal anesthesia (CCA) is an extremely rare and challenging condition causing neurotrophic keratitis in pediatric population. It can occur in isolation or be associated with congenital abnormalities affecting the mesoderm, ectoderm, or brainstem. In 1984, Rosenberg classified patients with congenital trigeminal anesthesia into three groups: (1) patients with isolated congenital trigeminal anesthesia; (2) patients with corneal and facial anesthesia associated with congenital abnormalities affecting the mesoderm; and (3) patients with CCA and associated focal brain stem abnormalities and without somatic malformations [4]. In corneal anesthesia, punctate keratitis and epithelial defects may occur spontaneously requiring urgent recognition and treatment to prevent from further progression to corneal ulcer. It is important to be aware about the possibility of this condition manifestation in childhood in order to prevent early visual loss. CCA requires constant supervision by an ophthalmologist. Detailed neurological, gastroenterological, nephrological, and gynecological examination should be performed to identify possible concomitant pathologies.

## 2. Case Report

We present a case of a six-year-old girl with systemic and neurological disorders and neurotrophic keratitis which is caused by congenital bilateral corneal anesthesia. The patient was born at 40 weeks’ gestation. The pregnancy was uncomplicated, mother only had herpes labialis during pregnancy at the third and fifth months. After birth, the positive-pressure ventilation was provided; hypotonia, hypertension of the torso extensors, leaned back head, lethargy and bilateral deafness were observed. A gastrostomy tube was formed due to dysphagia. At the age of one year and five months, cochlear implant surgery was performed. Nevertheless, the patient is deaf, she is not able to walk and talk, and contact is maintained through gestures and vision. There are no known hereditary diseases in the family, parents are healthy.

**Figure 1:**
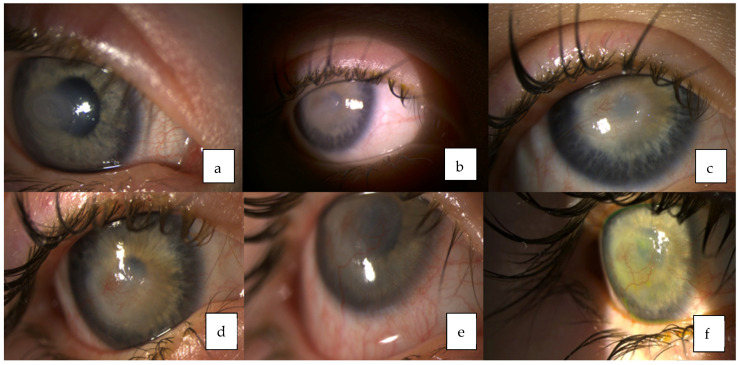
Dynamic of the right eye corneal ulcer: (**a**) Corneal ulcer. (**b**) Right eye after amniotic membrane transplantation. (**c**–**e**) Corneal opacity and neovascularization. (**f**) Right eye after anti-VEGF subconjunctival injection.

The patient presented to the Department of Ophthalmology at the Hospital of Lithuanian University of Health Sciences, Kaunas clinics at two years of age due to the right eye corneal erosion that was unresponsive to treatment. The patient’s right eye was treated with antibiotic drops, artificial tears and ointments, regenerating agent (Cacicol). Despite intensive topical treatment, only a slight improvement was observed. Later severe inflammation associated with hypopyon and iris hyperemia developed in the right eye. Intraocular pressure was normal in both eyes. Corneal scrapes microscopy, cytology, and culture of the right eye did not show any infectious agents. Since the corneal defect was unresponsive to conservative treatment, amniotic membrane transplantation was performed. The corneal ulcer epithelialized. However, a vascularized central corneal opacity has formed (Figure 1).

**Figure 2:**
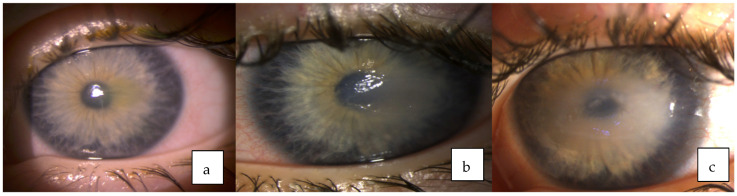
Dynamic of the left eye: (**a**) Central corneal erosion. (**b**) Superficial corneal ulcer. (**c**) Stromal corneal ulcer.

A year later patient scratched her left eye with the nail and presented at the department of ophthalmology due to the left eye corneal erosion. The patient was treated with antibiotic drops, anti-inflammatory drugs, artificial tears, ointments, therapeutic contact lens and regenerating agent (Cacicol) but all of the means were effective only for a short time and erosion progressed to the stromal corneal ulcer with surrounding corneal edema and iris hyperemia (Figure 2).

**Figure 3:**
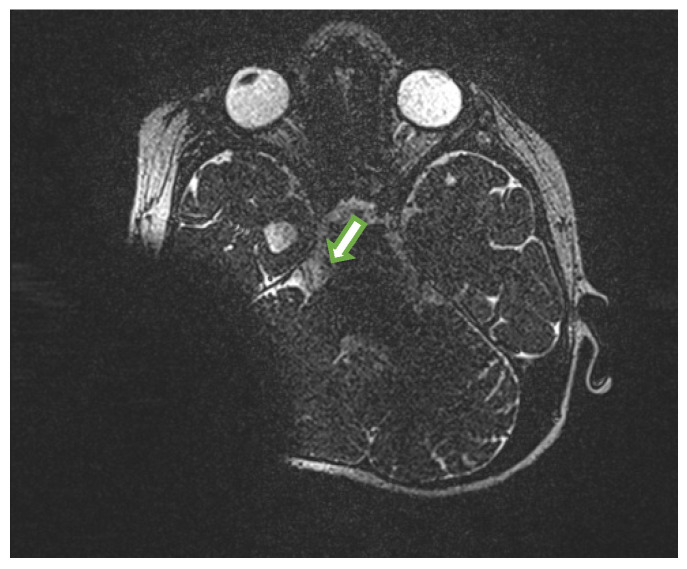
MRI of the patient. The arrow shows hypoplastic trigeminal nerve on the right side (MRI defect due to the cochlear implant).

Investigation and diagnosis: The patient carries a normal female karyotype (46,XX) and no significant alterations in chromosomes structure were discovered. Nevertheless, the taken samples are under further investigation by geneticists abroad to determine the cause of her systemic disorder. The patient has dysplastic facial features: expressed metopic suture, small lower jaw, high palate, facial amimia, and large ears. Corneal sensitivity and sensation in the distribution of the trigeminal nerve were not assessed due to cognitive function impairment and general patient’s condition. A detailed ocular examination was carried out to rule out all possible secondary causes of corneal aesthesia. A very rare blinking rate (once per minute) was observed. Corneal scrapings for culture were repeated during the exacerbation period. However, no bacterial or fungal growth was identified, and a test for HSV was negative as well. Brain MRI showed that both cisternal segments of the trigeminal nerves and parts in Meckel’s cave were small in diameter and hypoplastic. In the right-side pontocerebellar corner one cranial nerve was differentiated (more likely VIII, VII was not clearly differentiated) and in the left side two cranial nerves (more likely to be VII and hypoplastic VIII) (Figure 3). Cisternal segments of the VI cranial nerve were well differentiated. The patient was diagnosed with congenital bilateral corneal anesthesia secondary to bilateral trigeminal hypoplasia and bilateral neurotrophic keratopathy.

**Figure 4:**
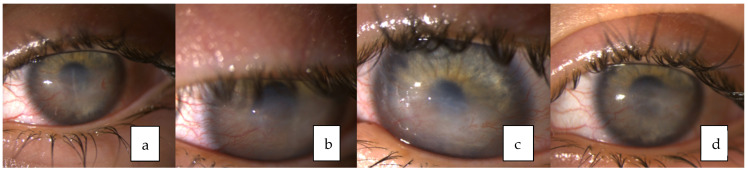
(**a**–**c**) Left eye weekly and (**d**) eight weeks after starting treatment with Cenegermin eye drops.

**Figure 5:**
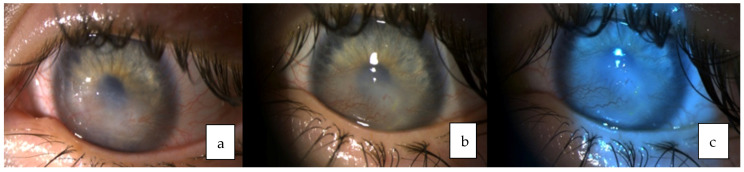
(**a**) Left eye before anti-VEGF subconjunctival injection. (**b**,**c**) Left eye after three anti-VEGF subconjunctival injection.

Treatment: Since vascularized corneal opacity formed after amniotic membrane transplantation in the right eye, different treatment options were considered. Recombinant human nerve growth factor (Cenegermin) was prescribed in accordance with FDA approval for its use in pediatric population. The course of one eye drop administered topically, six times daily for eight weeks was started. Gradual improvement was observed and the corneal epithelium defect healed. However, severe corneal opacity with neovascularization formed (Figure 4). The case was presented to the European Reference Network-Eye Diseases virtual clinic for the discussion about the treatment options for corneal neovascularization in the presence of CCA. Treatment with anti-VEGF subconjunctival injection to inhibit further corneal neovascularization was suggested. Three injections with three-months interval have already been performed. The corneal neovascularization remains stable (Figure 5). Maintenance therapy with artificial tears every hour during the day and ointment at night are prescribed. The patient is still under monthly supervision by an ophthalmologist.

## 3. Discussion

CCA usually presents in early childhood between the age of 8 and 12 months (when the time of a child being awake increases). However, a case of unilateral hypoplasia of the trigeminal ganglion has been reported in a 31-year-old women [5]. CCA can be easily misdiagnosed in favor of more common neurotrophic keratitis causes such as HSV or HZV keratitis, dry eye syndrome or recurrent epithelial erosions. It is usually bilateral disorder, although unilateral cases have been reported [5,6,7]. CCA tends to be sporadic. However, Purcell John. J. et al. described a case of a family in which 13 of 25 family members had isolated congenital corneal hypesthesia and in six of them epithelial erosion was found [8]. CCA can be isolated [9] or included in complex syndromes as well. Rosenberg reviewed 43 cases and classified congenital trigeminal anesthesia into three groups [4]. Considering this classification, the patient we presented does not match to any of these groups. There are more cases of CCA reported in the literature that are associated with other disorders [4] but none of them is similar to ours. There is a suspicion of glycoprotein metabolism defect due to psychomotor development disorder and dysmorphic facial features. However, neurotrophic keratopathy or hypoplasia of V, VII, VIII cranial nerves is not associated with clinical expression of glycoprotein metabolism disorder. There is a possibility that the patient has multiple diseases, since only a part of the patient’s clinical signs may be due to glycoprotein metabolism disorder. We are not aware of any condition in which brain stem dysfunction, such as deafness and dysphagia, congenital trigeminal anesthesia, and glycoprotein metabolism disorder are presented together.

It is essential to exclude acquired causes of corneal anesthesia, before the diagnosis of CCA is confirmed. Trigeminal nerve impairment can result from space occupying lesions of the cerebellopontine angle or diseases of the coronal sinus. Ocular causes of acquired corneal anesthesia include keratitis due to HSV or HZV. Orbital apex lesions, such as tumors or pseudotumor, could involve the ophthalmic branch of the trigeminal nerve. Systemic diseases, such as diabetes, vitamin A deficiency or multiple sclerosis can be a cause of impaired corneal sensitivity as well [10].

The first signs of CCA can be similar to conjunctivitis, such as recurrent episodes of red eye and discharge without pain [11]. The main corneal involvements are superficial keratopathy, persistent epithelial defects, and corneal ulcers that may lead to corneal perforation or infectious keratitis [12]. Functional and morphological examinations are required to provide insight into the pathogenesis of neurotrophic keratopathy. Functional examination includes evaluation of corneal sensation with a Cochet-Bonnet esthesiometer, whereas corneal confocal microscopy is used for morphological examination, although confocal microscopy is difficult to perform in children. Detailed examination including blood tests, roentgenography, abdominal and renal ultrasonography, and neurological examination should be performed to identify any possible systemic associations.

Medical treatment usually consists of artificial tears and ointments, autologous serum eyedrops and topical antibiotics when necessary. Surgical treatment options, which include tarsorrhaphy, conjunctival flap and amniotic membrane transplantation, are applied to corneal ulcers not responding to conservative treatment. Tarsorrhaphy is the most commonly used method to promote corneal healing [13]. However, in our case it was not considered since the patient is not able to talk, she does not hear, and she only communicates with gestures and eyesight.

According to some reports, a complete corneal healing was achieved in 90% of patients during an average of 18.4 days, after amniotic membrane transplantation in the corneal epithelial defects or ulcers caused by neurotrophic keratitis, with more than two lines improved visual acuity in 52.3% of patients [14]. Amniotic membrane transplantation was performed in the right eye of our patient, however, corneal opacity that impaired vision has developed. Corneal transplantation has a poor prognosis in CCA. According to the literature, scarring recurred or non-transparent scar developed in the grafts [11,15]. In recent years, new treatment strategies and methods have been developed. Regenerating agent (RGTA, Cacicol) is a matrix containing large polymers mimicking heparan sulfates. In a retrospective study corneal ulcers, which were unresponsive to conventional therapy, healed after usage of Cacicol in 9 cases out of 11 [16]. A recombinant nerve growth factor (Cenegermin, Oxervate) has the effect on epithelial proliferation and improved sensory functions of the cornea for patients with neurotrophic keratitis [17]. The effectiveness of Oxervate was confirmed in two main studies involving 204 patients with moderate (persistent epithelial defect) or severe (corneal ulcer) neurotrophic keratitis. In the first study, 74% of the patients treated with Oxervate achieved complete healing of the eye’s surface compared with 43% in vehicle-treated patients group [18]. In the second study, healing of the cornea was observed in 70% in the Oxervate group compared to 29% in the vehicle-treated group [19]. Better treatment results are obtained when treating only a corneal epithelial defect rather than an advanced corneal ulcer. In the case we presented the corneal ulcer healed. However, corneal opacity remained and severe neovascularization of the cornea occurred. The reason why the corneal opacity remained could be that we had to wait about two months until the beginning of the treatment (due to the high cost of Cenegermin, difficulties in receiving medication and the approval of Bioethics Committee to prescribe it for a child). Although it is permitted to use for children and adults in the United States, in Europe this treatment is approved only for adults.

Cenegermin efficacy and safety has been already demonstrated in a couple of pediatric cases [20,21,22]. Complete healing of the corneal epithelium defect was observed in a 3-year-old boy with neurotrophic keratitis due to rhabdomyosarcoma surgery of the jaw [22]. The healing of the persistent corneal epithelial defect, the resolution of the opacity and the improvement of corneal sensitivity were observed in a nine-year-old patient with pontine tegmental cap dysplasia [20]. Complete healing was achieved in both eyes after Cenegermin use for bilateral neurotrophic keratopathy in CCA: during the 16 months follow-up, no recurrence was noticed and corneal opacities gradually became clearer. However, the improvement in corneal sensitivity was insignificant [21]. Corneal sensitivity did not improve in our case, most probably due to the congenital profound sensory innervation deficit.

Common side effects of Cenegermin are eye pain and redness, increased lacrimation, eyelid pain, foreign body sensation, and corneal neovascularization. In none of the reported cases adverse events were observed. In case we presented severe neovascularization of the cornea occurred. Corneal neovascularization could be a side effect of the Oxervate or it could be the result of a delayed treatment.

The usage of subconjunctival anti-VEGF injections in order to prevent corneal neovascularization, shows promising outcomes. The results of a randomized control trial showed a reduction in the mean area of corneal neovascularization by 36% in the 15 eyes treated with bevacizumab compared with an increase of 90% in eyes that received placebo [23]. However, it is more useful in early stage of neovascularization and has weaker effect in cases of mature corneal neovascularization [24].

## 4. Conclusions

To conclude, neurotrophic keratitis due to CCA can be a cause of severe vision impairment in childhood. Various treatment approaches should be considered in order to achieve the best possible results. Similar treatment results were obtained in both eyes with two different treatment methods: amniotic membrane transplantation and recombinant human nerve growth factor. A combination of various factors, such as the stage of neurotrophic keratitis at the beginning of the treatment, the underlying cause, patient’s general condition and availability of medication contributes to the final treatment outcome.

## Data Availability

Not applicable.

## References

[1] Shaheen B.S., Bakir M., Jain S. (2014). Corneal nerves in health and disease. Surv. Ophthalmol..

[2] Dua H.S., Said D.G., Messmer E.M., Rolando M., Benitez-del-Castillo J.M., Hossain P.N., Shortt A.J., Geerling G., Nubile M., Figueiredo F.C. (2018). Neurotrophic keratopathy. Prog. Retin. Eye Res..

[3] Sacchetti M., Lambiase A. (2014). Diagnosis and management of neurotrophic keratitis. Clin. Ophthalmol..

[4] Rosenberg M.L. (1984). Congenital trigeminal anaesthesia: A review and classification. Brain.

[5] Milne A.D., Chili L., Mishra A.V., Maxner C.E. (2005). Unilateral hypoplasia of the trigeminal ganglion. Can. J. Ophthalmol..

[6] Morishige N., Morita Y., Yamada N., Nishida T., Sonoda K.H. (2014). Congenital hypoplastic trigeminal nerve revealed by manifestation of corneal disorders likely caused by neural factor deficiency. Case Rep. Ophthalmol..

[7] Voyatzis G., Mukherjee A., Rajan M.S., Allen L.E. (2012). Congenital unilateral corneal anaesthesia with microphthalmos: A case report. Case Rep. Ophthalmol. Med..

[8] Purcell J.J., Krachmer J.H. (1979). Familial corneal hypesthesia. Arch. Ophthalmol..

[9] Hashmi S.J., Chow G., Bittner S.B., Rittey C.D., Williams L.H.P. (2004). Congenital trigeminal anaesthesia. Dev. Med. Child Neurol..

[10] Semeraro F., Forbice E., Romano V., Angi M., Romano M.R., Filippelli M.E., Di Iorio R., Costagliola C. (2014). Neurotrophic keratitis. Ophthalmologica.

[11] Clarke M.P., Sullivan T.J., Kobayashi J., Rootman D.S., Cherry P.M.H. (1992). Familial congenital corneal anaesthesia. Aust. N. Z. J. Ophthalmol..

[12] Ramaesh K., Stokes J., Henry E., Dutton G.N., Dhillon B. (2007). Congenital corneal anesthesia. Surv. Ophthalmol..

[13] Versura P., Giannaccare G., Pellegrini M., Sebastiani S., Campos E.C. (2018). Neurotrophic keratitis: Current challenges and future prospects. Eye Brain.

[14] Mead O., Tighe S., Tseng S. (2020). Amniotic membrane transplantation for managing dry eye and neurotrophic keratitis. Taiwan J. Ophthalmol..

[15] Anseth A. (1968). Congenital bilateral corneal anesthesia. Acta Ophthalmol..

[16] Salazar-Quiñones L., Molero-Senosiáin M., Aguilar-Munoa S., Gegúndez-Fernández J.A., Díaz-Valle D., Muñoz-Hernández A.M., Benítez-Del-Castillo J.M. (2020). Management of corneal neurotrophic ulcers with Cacicol®-RGTA (ReGeneraTing Agent): A case series. Arch. Soc. Española Oftalmol. (Engl. Ed.).

[17] Clinical Review of Oxervate. https://www.accessdata.fda.gov/drugsatfda_docs/nda/2018/761094Orig1s000MedR.pdf.

[18] Bonini S., Lambiase A., Rama P., Sinigaglia F., Allegretti M., Chao W., Mantelli F., Bonini S., Lambiase A., Rama P. (2018). Phase II randomized, double-masked, vehicle-controlled trial of recombinant human nerve growth factor for neurotrophic keratitis. Ophthalmology.

[19] Pflugfelder S.C., Massaro-Giordano M., Perez V.L., Hamrah P., Deng S.X., Espandar L., Foster C.S., Affeldt J., Seedor J.A., Afshari N.A. (2020). Topical Recombinant Human Nerve Growth Factor (Cenegermin) for Neurotrophic Keratopathy. Ophthalmology.

[20] Fausto R., Ceccuzzi R., Micheletti E., Clerici R., Riva I., Katsanos A., Oddone F., Quaranta L. (2020). A case report of pediatric neurotrophic keratopathy in pontine tegmental cap dysplasia treated with cenegermin eye drops. Medicine.

[21] Leto M.G., Toro M.E., Indemini P.E., Fruttero C., Denina M., Dalmazzo C., Sannia A., Vaiano A.S. (2021). Pediatric use of recombinant human nerve growth factor 20 μg/mL eye drops (cenegermin) for bilateral neurotrophic keratopathy in congenital corneal anesthesia. Cornea.

[22] Pedrotti E., Bonetto J., Cozzini T., Fasolo A., Marchini G. (2019). Cenegermin in Pediatric Neurotrophic Keratopathy. Cornea.

[23] Petsoglou C., Balaggan K.S., Dart J.K.G., Bunce C., Xing W., Ali R., Tuft S.J. (2013). Subconjunctival bevacizumab induces regression of corneal neovascularisation: A pilot randomised placebo-controlled double-masked trial. Br. J. Ophthalmol..

[24] Krizova D., Vokrojova M., Liehneova K., Studeny P. (2014). Treatment of Corneal Neovascularization Using Anti-VEGF Bevacizumab. J. Ophthalmol..

